# Plant-parasitic nematodes on hemp in the Pacific Northwest of the United States

**DOI:** 10.1186/s42238-025-00301-y

**Published:** 2025-07-16

**Authors:** Lester A. Núñez-Rodríguez, Hannah M. Rivedal, Cynthia M. Ocamb, David H. Gent, Inga A. Zasada

**Affiliations:** 1 Department of Botany and Plant Pathology, Oregon State University, 2701 SW Campus Way, Corvallis, OR 97331 USA; 2 Forage Seed and Cereal Research Unit, USDA-ARS, 3450 SW Campus Way, Corvallis, OR 97331 USA; 3 Horticultural Crops Disease and Pest Management Unit, USDA-ARS, 3420 Orchard Ave, Corvallis, OR 97330 USA

**Keywords:** Hemp, Identification, Plant-parasitic nematodes, *Pratylenchus* spp

## Abstract

**Background:**

Plant-parasitic nematodes are one of the most important biotic factors that impact crop production globally. Since hemp cultivation in the U.S. was banned from 1970 to 2018, little information is available about current plant-parasitic nematode pressure on U.S. hemp production. The production of hemp has gained interest in Washington and Oregon, states where several genera of plant-parasitic nematodes have been associated with various crops. This report is the first to define plant-parasitic nematodes associated with hemp in these states in the Pacific Northwest.

**Methods:**

Soil and root samples from hemp fields were collected in early autumn in 2021, 2022, and 2023. The occurrence, population density, and identity of plant-parasitic nematodes in these samples were determined using morphological and molecular identification methods. A Bayesian analysis of available sequence data was used to analyze phylogenetic relationships of nematode species found in hemp fields. Additionally, the host status of hemp ‘Alpha Explorer’ to three plant-parasitic nematodes, *Meloidogyne chitwoodi*, *M. hapla*, and *Pratylenchus neglectus*, was tested under greenhouse conditions. The occurrence of plant-parasitic nematodes and reproduction factor (final population density/initial population density) values of the three nematode species were analyzed with non-parametric methods.

**Results:**

*Pratylenchus* spp. were the most frequent plant-parasitic nematodes recovered from soil samples, being present in ~ 63% of samples (*n* = 107). Only two endoparasitic nematodes, *Meloidogyne* spp. and *Pratylenchus* spp., were found in root samples, with *Pratylenchus* spp. as the most frequent (20 out of 24 fields). A large diversity of *Pratylenchus* spp. was detected in hemp root samples. Hemp ‘Alpha Explorer’ was a poor host for *P. neglectus*, resulting in low reproduction values (< 1). Additionally, results of our study indicated that hemp is not a host for *M. chitwoodi*.

**Conclusions:**

*Pratylenchus* spp. were the most frequent plant-parasitic nematodes found in hemp fields in Oregon and Washington. This study reports for the first time five *Pratylenchus* species (*Pratylenchus crenatus*,* P. fallax*,* P. hexincisus*,* P. neglectus*, and *P. scribneri*) associated with hemp in Oregon and Washington; *P. penetrans* was also found in the region on hemp. The host status results indicate that hemp can be considered a non-host for *M. chitwoodi* and a poor host for *M. hapla* and *P. neglectus*.

**Supplementary Information:**

The online version contains supplementary material available at 10.1186/s42238-025-00301-y.

## Introduction

Hemp (*Cannabis sativa*), an herbaceous annual crop, is one of two crops from the Cannabaceae family cultivated in the Pacific Northwest (PNW; Oregon and Washington) of the United States (Punja 2021). Hemp cultivation in the United States has been historically for fiber production. However, the 1937 Marihuana Tax Act classified all hemp crops as marijuana. Therefore, subject to the new drug enforcement laws with the Controlled Substance Act of 1970, hemp cultivation was banned, regardless of end use (Cranshaw et al. 2019). It was not until the passage of the 2014 Farm Bill (U.S. H.R. 2642 - Agricultural Act of 2014 113th Congress [2013–2014]) that hemp was included in pilot research programs to reincorporate this crop into agriculture systems of the U.S. The 2018 Farm Bill removed hemp and hemp seeds from the Drug Enforcement Administration’s schedule of controlled substances, defining hemp as a hemp plant or any part of such plant with a maximum concentration of delta-9 tetrahydrocannabinol (THC) of 0.3% in terms of dry weight (Congress 2018).

After the 2014 and 2018 Farm Bills, the production of hemp in the U.S. tripled from 10,406 ha in 2017 to 31,637 ha in 2018. In 2019, the hemp acreage reached a maximum peak of 81,393 ha (Vote, Hemp 2021). After this production peak, hemp acreage decreased to a total of 11,458 ha in 2022. Hemp acreage in Oregon and Washington in 2022 was 850 ha and 152 ha, respectively (USDA-NASS 2023). Although hemp is grown for different purposes in the PNW, the industry is dominated by outdoor floral hemp production for essential oil extraction valued at ~ $93 million (USDA-NASS 2023). Because hemp has only been widely produced recently in the U.S., there is little information about the biotic challenges hemp growers face, including plant-parasitic nematodes (Bernard et al. 2022). These organisms are estimated to cause more than $170 billion in economic losses to overall crop production globally per year (Kantor et al. 2022).

A study conducted in Florida, a state with a subtropical climate, reported seven different genera of plant-parasitic nematodes, *Nanidorus*, *Rotylenchulus*, *Helicotylenchus*, ring nematode (*Criconemella* or *Mesocriconema*), *Belonolaimus*, *Tylenchorhynchus*, and *Meloidogyne*, associated with hemp (Desaeger et al. 2023). *Pratylenchus* spp. have also been reported in hemp (Bernard et al. 2022; Núnez-Rodríguez et al. 2023), as well as *Meloidogyne* spp. (Nunez-Rodriguez et al. 2024). Most of these nematodes are present in the PNW of the U.S. with *Pratylenchus* and *Meloidogyne* being as the most common genera (Zasada et al. 2019). Despite the recent interest of hemp as a new crop in the region, no studies have been conducted to identify plant-parasitic nematodes in this crop or to evaluate the host status of hemp for plant-parasitic nematodes of concern. This study aimed to provide information on the presence of plant-parasitic nematodes in hemp fields in Oregon and Washington and determine the host status of hemp to three plant-parasitic nematodes commonly found in the PNW, *Meloidogyne chitwoodi*,* M. hapla*, and *Pratylenchus neglectus*.

## Materials and methods

### Sample collection

Composite soil samples from hemp fields were collected towards the end of the growing season (September) in 2021, 2022, and 2023, just before commercial harvest of the plants occurred in late September to early October. One or two 100-plant transects (~ 70 cm between plants) per field were selected for sampling based on field size, and 10 soil cores were collected per transect. In fields with one transect, one composite soil sample was collected, whereas two composite soil samples were collected in fields with two sampling transects. Soil sampling was performed using a 2.5-cm-diameter soil probe with a probe length of 30 cm (12” L soil sampler; JMC, Newton, IA). Cores were collected from every 10th plant in each transect and as close to the base of the plant crowns as possible. The 10 cores collected per transect were mixed in a bucket and then placed into a labeled plastic bag. In 2022 and 2023, root sampling was allowed by hemp growers. Roots were collected using a shovel. When possible, hemp roots were collected from the same plants near where soil samples were collected. When growers were concerned about potential damage caused by root sampling, only five plants out of the 10 were sampled. Samples were stored in a cooler during transport and then stored in at 4° C until processing.

### Nematode extraction and enumeration

Soil samples (*n* = 45) collected in 2021 were extracted using a semi-automatic elutriator followed by sugar centrifugation with a 1 M sugar solution to recover vermiform nematodes from 250 g of wet soil (East et al. 2019). At the time of processing, fine roots (*n* = 42, no roots were obtained from three fields) recovered on the upper sieve when elutriating were collected to extract nematodes using the Baermann funnel method (Ingham 1994), with the main goal of generating preliminary information on the occurrence of plant-parasitic nematodes in hemp roots and to justify future requests to growers for hemp root collections. Soil samples collected in 2022 (*n* = 44) and 2023 (*n* = 18) were decant-sieved followed by sugar centrifugation with a 1 M sugar solution (Jenkins 1964). Root samples from Washington (*n* = 7 collected in 2022) and Oregon (*n* = 10 and *n* = 7 collected in 2022 and 2023, respectively) were washed, cut into 1–2 cm long pieces, mixed, and placed under intermittent mist for nematode extraction for seven days (Zasada et al. 2015). Plant-parasitic nematodes were enumerated and identified to the genus level based on morphological features using an inverted microscope. Frequency of occurrence of plant-parasitic nematodes in soil was analyzed for effects using χ^2^ analysis in RStudio v4.3.1 (R Core Team 2023).

### Molecular identification of plant-parasitic nematodes

Three *Pratylenchus* populations recovered from fine roots collected in 2021 were selected for molecular identification using species-specific primers from the *β*-1,4-endoglucanase gene region. Eight nematodes from each population were hand-picked and cut prior to DNA extraction (Peetz and Zasada 2016); each nematode represented a soil sample. Fifteen *Pratylenchus* populations (eight and seven populations from Oregon and Washington, respectively) obtained from the 2022 hemp roots were identified via molecular techniques. The partial gene cytochrome oxidase I (*COX1*) was used for identification, which was amplified using the primers JB3 (5’- TTTTTTGGGCATCCTGAGGTTTAT − 3’)/JB4.5 (5’- TAAAGAAAGAACATAATGAAA ATG − 3’) (Bowles et al. 1992), F7bp (GGDTGRACWTTHTAYCCNCC − 3’)/B4.5 (Bowles et al. 1992; Ozbayrak et al. 2019), or COIFGED (5’- CCTTTGGGCATCCNGARGTNTAT − 3’)/B5GED (5’- ACCTAAACTTARWACRTARTGAAAATG − 3’) (Bowles et al. 1992; Ren et al. 2024). Only one set of primers was used per population. Samples were sent for bidirectional sequencing to the Oregon State University Center for Quantitative Life Science (Corvallis, OR). Sequence identification was determined using the NCBI Basic Local Alignment Search Tool (BLAST).

Reference sequences of *Pratylenchus* spp. were retrieved from GenBank to estimate phylogenetic relationships. The algorithm Clustal W was used for sequence alignment (Thompson et al. 1994) and trimmed using BioEdit v.7.0.5.3 (Hall 1999). jModelTest 2.1.10 v20160303 was used to select the best substitution model (Darriba et al. 2012) to perform the phylogenetic analyses using a Bayesian analysis method (Larget and Simon 1999). Finally, the phylogenetic tree was visualized using FigTree v1.4.3 (Rambaut 2016). Sequences obtained in this study were deposited to GenBank (accession numbers PQ203080- PQ203087, PQ212514-, PQ212522, PQ218872- PQ218880, PQ219697, and PQ220377- PQ220379).

### Host status of hemp for plant-parasitic nematodes

The pathogenicity of *Meloidogyne hapla* (reported in Núñez-Rodríguez et al. 2024), M. *chitwoodi* and *Pratylenchus neglectus* were tested under greenhouse conditions in 2023. Briefly, eggs of *Meloidogyne* spp. were extracted from pure greenhouse cultures using the NaOCl method (Hussey and Baker 1973), whereas *P. neglectus* was obtained from pure carrot disc cultures (Coyne et al. 2014). The *M. hapla* population was originally collected from a vineyard in Alderdale, WA. The *M. chitwoodi* population was originally collected from a potato field in Prosser, WA. The *P. neglectus* population was originally collected from a wheat field in Pendelton, OR. All nematodes were molecularly identified to the species level by the North Carolina Department of Agriculture and Consumer Services based upon sequencing of the internal transcribed spacer (ITS) region (Raleigh, NC). Twenty 3-L plastic pots were filled with 2.1 kg of steam-pasteurized 1:1 sand/loam soil mixture. One five-week-old hemp ‘Alpha Explorer’ seedling was transplanted into each pot. Five pots per nematode species were inoculated with 4,000 eggs (*M. chitwoodi* and *M. hapla*) or 2,000 nematodes (mix of juveniles and adults of *P. neglectus*), and the five remaining pots were left non-inoculated. Three positive controls for each nematode species, tomato ‘Rutgers’ for *Meloidogyne* spp. and wheat ‘Yuma’ for *P. neglectus*, were planted in 0.6-L plastic pots filled with 0.4 kg of the soil mixture. Positive control tomato plants were inoculated with 1,100 *Meloidogyne* eggs per nematode species and positive control wheat plants were inoculated with 500 *P. neglectus*. The experimental design in this experiment was a completely randomized design. The average greenhouse temperature after inoculation was 20.8 ± 3.2°C. Plants were harvested 60 days after inoculation. *Meloidogyne* spp. were extracted from roots using the NaOCl method (Hussey and Baker 1973), while *P. neglectus* was extracted from roots and soil using the intermittent mist and Baermann funnel methods, respectively, as described above. The reproduction factor (RF = final population density/initial population density) was calculated to determine the host status of hemp to these nematodes. The experiment was conducted twice.

The RF of each trial was analyzed separately since RF values of plant-parasitic nematodes on hemp did not meet the assumptions of normality (Shapiro test) and homoscedasticity (Levene test). Data was analyzed using the non-parametric Kruskal-Wallis and Mann-Whitney tests in RStudio v4.3.1 (R Core Team 2023).

## Results

### Occurrence of plant-parasitic nematodes in hemp fields

A total of 107 soil samples (77 from Oregon and 30 from Washington) were processed, representing 66 hemp fields (50 from Oregon and 16 from Washington), from which seven different plant-parasitic nematodes were identified. The endoparasitic nematode *Pratylenchus* spp. were the most frequently detected nematodes, present in ~ 63% of the samples, followed by the ectoparasitic nematodes, *Paratylenchus* and *Tylenchorhynchus* (Table 1; *P* < 0.05). Four other plant-parasitic nematodes were detected at < 5% occurrence, including Criconematidae, *Helicotylenchus*,* Meloidogyne*, and *Xiphinema.* There were no statistical differences observed when the frequency of occurrence of plant-parasitic nematodes was analyzed by state or combined over both states (Supplementary Table 1; *P* > 0.05).

**Table 1:** Frequency of occurrence (FO%), maximum population density (Max.), and mean population density (nematodes/250 g of soil) of plant-parasitic nematodes when present in soil samples from hemp fields in the Pacific Northwest (Oregon and Washington) collected in September of 2021 and 2023

Plant-parasitic nematodes	FO%*	Max.	Mean
Criconematidae	3.7 a	27	9
*Helicotylenchus*	3.7 a	20	10
*Meloidogyne*	3.7 a	26	20
*Paratylenchus*	22.4 b	12,325	988
*Pratylenchus*	62.6 c	228	338
*Tylenchorhynchus*	16.8 b	320	37
*Xiphinema*	3.7 a	27	15

*Frequency of occurrence data were analyzed for effects using χ^2^ analysis. Values followed by the same letter in the same column are not significantly different from each other (*P* < 0.05)

*Pratylenchus* spp. were the only endoparasitic nematodes obtained from the fine root samples collected in 2021, present in 30 out of the 42 samples (results not shown). In 2022, root samples were collected from 17 fields; 10 fields in Oregon and 7 fields in Washington. In Washington, *Pratylenchus* spp. were the only endoparasitic nematodes found in hemp root samples detected in all fields with population densities ranging from 1,195 to 13,362 nematodes in 100 g of wet root (Table 2). Two genera of endoparasitic nematodes were found in root samples collected in 2022 in Oregon, *Pratylenchus* spp. and *Meloidogyne* spp. *Pratylenchus* spp. were found in all 10 fields and three of these fields also contained *Meloidogyne* spp. The population densities of *Pratylenchus* and *Meloidogyne* ranged from 43 to 6,461 nematodes and 6 to 21,336 s-stage juveniles (J2) in 100 g of wet root, respectively (Table 2). Nematodes obtained in 2022 were selected for species identification using molecular methods.

In 2023, *Pratylenchus* spp. and *Meloidogyne* spp. were the only endoparasitic nematodes found in the root samples collected in Oregon (Table 2). *Pratylenchus* spp. were detected in 3 of 7 fields with a range of population densities from 498 to 2,737 nematodes in 100 g of wet root. *Meloidogyne* spp. were detected in 1 of 6 fields with a population density of 296 J2 in 100 g of wet root.

**Table 2:** Population density of endoparasitic nematodes in roots expressed in 100 g of wet root obtained from hemp fields (*n* = 24)

Field	Year	State	*Pratylenchus* spp.	*Meloidogyne* spp.
WA1	2022	Washington	1,195	0
WA2	2022	Washington	5,071	0
WA3	2022	Washington	2,085	0
WA4	2022	Washington	13,362	0
WA5	2022	Washington	12,886	0
WA6	2022	Washington	5,040	0
WA8	2022	Washington	5,446	0
OR2	2022	Oregon	385	0
OR3	2022	Oregon	532	24
OR5	2022	Oregon	3,871	0
OR6	2022	Oregon	43	0
OR7	2022	Oregon	116	0
OR9	2022	Oregon	128	0
OR10	2022	Oregon	6,461	21,336
OR11	2022	Oregon	3,813	0
OR12	2022	Oregon	43	0
OR18	2022	Oregon	841	6
OR6	2023	Oregon	2,737	0
OR10	2023	Oregon	2,107	296
OR12	2023	Oregon	0	0
OR15	2023	Oregon	0	0
OR16	2023	Oregon	0	0
OR18	2023	Oregon	498	0
OR19	2023	Oregon	0	0

### Molecular identification of plant-parasitic nematodes

In 2021, three *Pratylenchus* spp. populations collected from Oregon were selected for molecular identification. These populations were identified as *Pratylenchus penetrans* (results not shown). In 2022, 15 populations of *Pratylenchus* spp. were molecularly identified, which included a *P. penetrans* population reported in Núñez-Rodríguez et al. (2023). In general, BLAST results ranged from 98.7 to 100% identity (Table 3). In Washington, four *Pratylenchus* spp. were identified: *P. hexincisus* (one sample out of seven), *P. neglectus* (five samples), *P. penetrans* (three samples), and *P. scribneri* (one sample). There were two samples with mixed *Pratylenchus* spp. One sample had three different *Pratylenchus* species (*P. hexincisus*,* P. neglectus*, and *P. scribneri*), whereas, the other sample contained two species (*P. neglectus* and *P. penetrans*). In Oregon, three *Pratylenchus* spp. were identified, *P. crenatus* (four out of eight samples), *P. fallax* (two samples), and *P. penetrans* (two samples) in root samples collected in 2022. No mixed *Pratylenchus* spp. populations were found in Oregon. Three samples had *Meloidogyne* spp. and one of these populations was identified as *Meloidogyne hapla* by Núñez-Rodríguez et al. (2024).

**Table 3:** Sequenced *Pratylenchus* spp. Populations recovered from hemp root samples collected in the Pacific Northwest (Oregon and Washington) including the top match percent identity based on the NCBI basic local alignment search tool

Population Code	Pratylenchus species	Accession number	Match in GenBank	% Identity
WA1	*P. hexincisus*	PQ220377	MK877488	98.7
WA1	*P. hexincisus*	PQ220378	MK877488	100
WA1	*P. hexincisus*	PQ220379	MK877485	100
WA1	*P. neglectus*	PQ212514	MK877772	99.8
WA1	*P. scribneri*	PQ203080	MZ203870	99.8
WA2	*P. neglectus*	PQ212515	MN366428	99.8
WA2	*P. neglectus*	PQ212516	MN366428	100
WA2	*P. neglectus*	PQ212517	MN366428	99.8
WA3	*P. penetrans*	PQ218872	MN453208	99.3
WA3	*P. neglectus*	PQ212518	MK877798	99.8
WA3	*P. neglectus*	PQ212519	MN366444	99.8
WA4	*P. neglectus*	PQ212520	MK877772	99.8
WA4	*P. neglectus*	PQ212521	MN366428	99.8
WA5	*P. penetrans*	PQ218873	MN453208	99.3
WA5	*P. penetrans*	PQ219697	MN453217	99.8
WA6	*P. neglectus*	PQ212520	MK877772	99.8
WA8*	*P. penetrans*	OP801849	MN453217	100
OR5	*P. fallax*	PQ218874	MN453209	100
OR6	*P. crenatus*	PQ203081	PP152242	99.8
OR7	*P. crenatus*	PQ203082	PP152242	99.8
OR9	*P. penetrans*	PQ218875	MN453208	99.5
OR9	*P. penetrans*	PQ218876	KY816942	100
OR9	*P. penetrans*	PQ218877	MN453208	99.8
OR9	*P. penetrans*	PQ218878	MT527066	100
OR10	*P. crenatus*	PQ203083	MK877465	99.5
OR10	*P. crenatus*	PQ203084	PP152242	99.5
OR10	*P. crenatus*	PQ203085	PP152242	99.8
OR10	*P. crenatus*	PQ203086	PP152242	99.8
OR11	*P. penetrans*	PQ218879	MK877988	99.1
OR12	*P. crenatus*	PQ203087	MK877465	99.7
OR18	*P. fallax*	PQ218880	MN453209	100

*Núnez-Rodríguez et al. (2023)

**Fig. 1:**
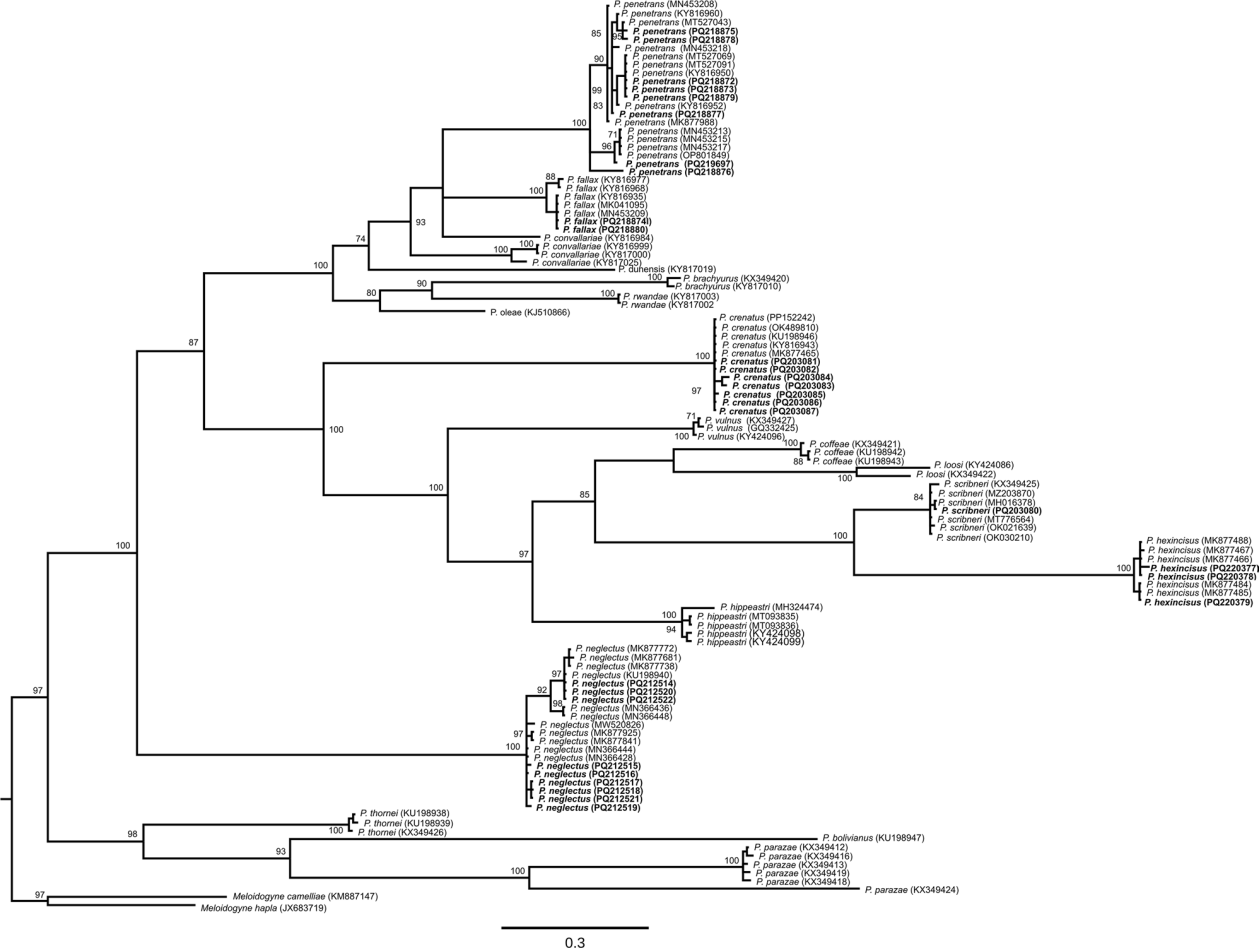
Phylogenetic relationships between *Pratylenchus* species populations collected from hemp fields in the Pacific Northwest (Oregon and Washington) as inferred from Bayesian analysis of the *COX1* gene sequences using the GTR + I + G model of nucleotide substitution. Posterior probabilities of over 70% are given for appropriate clades. Sequences generated in this study are highlighted in bold

*Pratylenchus penetrans* was found relatively frequently in samples collected from both states. However, except for *P. neglectus*, the other *Pratylenchus* spp. were not recovered from both states (Table 3). The phylogenetic analysis placed the *P. fallax* and *P. scribneri* populations in clades with *P. fallax* and *P. scribneri* sequences with posterior probability (PP) values of 100 and 84, respectively. All *P. fallax* and *P. penetrans* populations identified in this study were placed within the *Penetrans* group (PP = 100; Fig. 1). Sequences of *P. penetrans* were placed in a clade with other *P. penetrans* sequences (PP = 100), with *P. penetrans* haplotypes forming subclades within this clade (Fig. 1). Populations of *P. crenatus*, *P. hexincisus*, and *P. neglectus* were also placed in clades with sequences of their respective species (PP = 100).

**Host status of hemp for plant-parasitic nematodes**.

**Fig. 2:**
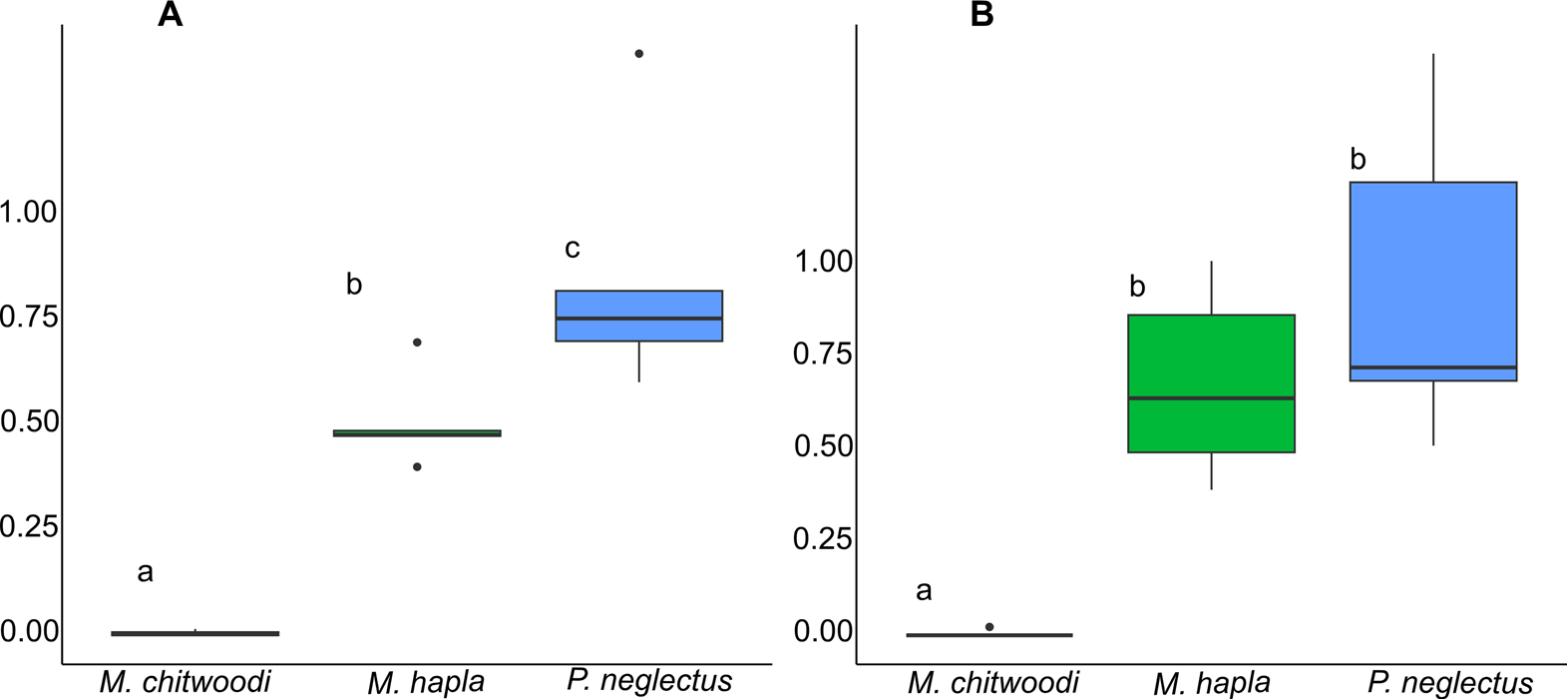
Reproduction factor (final population density/initial population density) of *Meloidogyne chitwoodi*, *M. hapla*, and *Pratylenchus neglectus* on hemp ‘Alpha Explorer’ under greenhouse conditions in two trials (A and B). Different letters above bars indicate a statistical difference according to the nonparametric tests Kruskal-Wallis and Mann-Whitney

Positive controls confirmed the viability of the nematode inoculum for both experiments. In these positive controls, the RF values (expressed as mean ± standard deviation) for *M. hapla* were 247.7 ± 17.7 and 169.4 ± 9.5 in the first and second trials, respectively. *Meloidogyne chitwoodi* had RF values of 118.9 ± 7.2 and 58.6 ± 10.7, whereas *P. neglectus* had RF values of 7.0 ± 3.5 and 8.4 ± 3.0 in the first and second trials, respectively. In both trials, *M. chitwoodi* had the lowest RF value (0.001 ± 0.006 and 0.004 ± 0.01 in first and second trials, respectively; *P* < 0.05). In the first trial, RF values for the three plant-parasitic nematodes were significantly different from each other (*P* = 0.002). However, in the second trial, RF values for *M. hapla* and *P. neglectus* were not statistically different (*P* > 0.05; Fig. 2). In both experiments, *M. hapla* and *P. neglectus* had RF values below 1 but not smaller than 0.5 (Fig. 2).

## Discussion

In 2014, hemp cultivation was allowed in the U.S. after being banned since 1970 (Johnson 2019). Therefore, there is limited information about factors that impact hemp production, including plant-parasitic nematodes. In the PNW, this is the first survey of plant-parasitic nematodes associated with hemp. Determining the occurrence and distribution of plant-parasitic nematodes is a crucial step towards determining the potential importance of these soilborne pathogens for crop production and future research efforts to manage nematodes (Zasada et al. 2010). All seven genera of plant-parasitic nematodes identified in soil samples in this study have been reported in the PNW of the U.S. as affecting different crops (Zasada et al. 2019). *Pratylenchus* was the most frequent genus recovered from both soil and hemp roots. This nematode is known to have a wide host range (> 2,000 plant species), including non-agricultural and economically important agricultural crops (Agrios 2005; Núñez-Rodríguez et al. 2022). This result is similar to what has been observed in the region across commodities, with *Pratylenchus* spp. being found in ~ 70% of samples considered in the PNW (Zasada et al. 2019).

Although *Meloidogyne* spp. are considered the most important plant-parasitic nematodes worldwide (Jones et al. 2013), *Meloidogyne* was not commonly recovered in this survey. This result is opposite to what has been reported by Desaeger et al. (2024) in Florida, where *Meloidogyne* spp. were the most frequently encountered plant-parasitic nematodes. This finding highlights the importance of region-specific sampling to assess the presence of plant-parasitic nematodes where little or no information is available.

Three samples from Oregon had both *Meloidogyne* and *Pratylenchus*, but one sample contained both nematodes with densities in the thousands: *Meloidogyne* sp. at 21,335 J2 and *Pratylenchus* sp. at 6,461 per 100 g of wet root (OR10 during 2022 in Table 2). The *Meloidogyne* population was identified as *M. hapla* (Núñez-Rodríguez et al. 2024). *Paratylenchus* spp. and *Tylenchorhynchus* spp. are two ectoparasitic nematodes that are commonly found in the PNW (Zasada et al. 2019). Both of these plant-parasitic nematodes were also commonly found in our study. There was a sample with an average of 12,325 nematodes per 250 g of soil (~ 149 *Paratylechus* spp. per g of soil). On pea, Upadhaya et al. (2019) demonstrated that an initial population of 4.5 *P. nanus*/g of soil caused a reduction of plant height, dry shoot weight, and seed weight. The population density we observed on hemp was 10 times higher than the initial population reported by Upadhaya et al. (2019).

The low occurrence of Criconematidae, *Helicotylenchus* spp., and *Xiphinema* spp. is in agreement with what has been reported in the PNW (Zasada et al. 2019). However, the low occurrence of these plant-parasitic nematodes does not mean that they do not pose a risk for hemp production. For example, *Xiphinema* spp. may be of concern for hemp growers due to the ability of this group of nematodes to transmit viruses that have been reported in hemp, such as Arabis mosaic virus (Harrison et al. 1974; McPartland 2000).

The partial *COX1* gene allowed for the identification of all 15 *Pratylenchus* spp. populations selected for this purpose, including the population found in OR10, which was identified as *P. penetrans* (Núñez-Rodríguez et al. 2024). The phylogenetic tree obtained in this study had a similar topology to the one reported in Janssen et al. (2017), which demonstrated the importance for this region to separate between closely related species such as *P. penetrans* and *P. fallax*.

The most frequent *Pratylenchus* spp. identified from hemp roots was *P. neglectus*, which was found in 10 samples (one sample from Oregon and nine from Washington), and *P. penetrans* in eight samples (five and three samples from Oregon and Washington, respectively). These species are two of the most import *Pratylenchus* species worldwide (Jones et al. 2013), including in the PNW region, where they and *P. crenatus* (another species identified in this study) can commonly be found associated with various crops (Zasada et al. 2019). *Pratylenchus fallax*,* P. hexincisus*, and *P. scribneri* were also identified in this study. This is the first time *P. fallax*,* P. scribneri*,* P. crenatus*, and *P. neglectus* have been reported on hemp in the PNW (Zasada et al. 2018). To our knowledge, this is the first time that hemp is reported to be a host for these nematodes. This is also the first report of *P. hexincisus* in the PNW or on hemp (Zasada et al. 2018).

The pathogenicity of *P. neglectus*, *M. hapla* (Núñez-Rodríguez et al. 2024)d *chitwoodi* (Zasada et al. 2019), the latter being a nematode of concern for potato growers in the PNW, was tested under greenhouse conditions. Hemp was a non-host for *M. chitwoodi* with RF values close to 0, whereas hemp was a poor host for *M. hapla* and *P. neglectus*. The poor host status of hemp for *M. hapla* was also observed by Coburn et al. (2024). However, reproduction of *M. hapla* has been reported to vary between hemp cultivars, from moderately susceptible to highly resistant (de Meijer 1993). In the case of *M. chitwoodi*, although not with the same hemp cultivar, our results agree with Kock et al. (1994), who reported hemp as non-host for this nematode.

This is the first research effort defining the occurrence, identity, and interaction of plant-parasitic nematodes on hemp in Oregon and Washington. Our study determined that *Pratylenchus* spp. were the most frequent plant-parasitic nematodes found across all hemp fields sampled. Additionally, five *Pratylenchus* species were found in hemp fields in the region. Based upon the host status of only one hemp cultivar, hemp seems to be a promising crop to consider for crop rotation systems in fields where *M. chitwoodi* is of concern, since it appears to be a non-host. Further studies should be conducted in microplot or field environments to evaluate the potential role of hemp to reduce population densities of *Meloidogyne* spp. and *Pratylenchus* spp. in the PNW.

## Electronic supplementary material

Below is the link to the electronic supplementary material.

Supplementary Material 1

## Data Availability

Data included in this paper can be shared upon request.
