# Supplementary material for: Plant-parasitic nematodes on hemp in the Pacific Northwest of the United States
Source: J Cannabis Res. 2025 Jul 16;7:47. doi: 10.1186/s42238-025-00301-y (PMC12265197; doi:10.1186/s42238-025-00301-y)
Supplement: Supplementary file 1 — Supplementary Material 1 [file 42238_2025_301_MOESM1_ESM.docx]

**SUPPLEMENTARY TABLES**

**Supplementary Table 1.** Frequency of occurrence (FO%*), maximum (Max.) population density (nematodes/250 g of soil), and mean population density (nematodes/250 g of soil) when present in a sample of plant-parasitic nematodes in Oregon, Washington, and the Pacific Northwest (Oregon and Washington).

| **Plant-parasitic nematodes** | **State/region** | | | | | | | | |
| --- | --- | --- | --- | --- | --- | --- | --- | --- | --- |
|  | **Oregon** | | | **Washington** | | | **PNW** | | |
|  | **FO%*** | **Max.** | **Mean** | **FO%*** | **Max.** | **Mean** | **FO%*** | **Max.** | **Mean** |
| *Criconemella* | 4.2a | 3 | 3 | 3.3 a | 27 | 27 | 3.7 a | 27 | 9 |
| *Helicotylenchus* | 4.2 a | 14 | 7 | 3.3 a | 20 | 20 | 3.7 a | 20 | 10 |
| *Meloidogyne* | 5.6 a | 26 | 20 | 0 | 0 | 0 | 3.7 a | 26 | 20 |
| *Paratylenchus* | 22.2 a | 12,325 | 1,442 | 26.7 a | 270 | 81 | 27 a | 12,325 | 988 |
| *Pratylenchus* | 55.6 a | 228 | 38 | 73.3 a | 190 | 41 | 62.6 a | 228 | 38 |
| *Tylenchorhynchus* | 12.5 a | 85 | 29 | 13.3 a | 320 | 99 | 16.8 a | 320 | 37 |
| *Xiphinema* | 5.6 a | 27 | 15 | 0 | 0 | 0 | 3.7 a | 27 | 15 |

*Frequency of occurrence data were analyzed for effects using χ2 analysis. Values followed by the same letter in the same column are not significantly different from each other (*P < 0.05*).
